# *Mycoplasma alkalescens* demonstrated in bronchoalveolar lavage of cattle in Denmark

**DOI:** 10.1186/1751-0147-49-2

**Published:** 2007-01-04

**Authors:** Branko Kokotovic, Niels F Friis, Peter Ahrens

**Affiliations:** National Veterinary Institute, New Technical University of Denmark, Bülowsvej 27, DK-1790 Copenhagen V, Denmark

**Keywords:** Mastitis, Mycoplasma Species, Bovine Respiratory Syncytial Virus, Standard Laboratory Procedure, Bulk Tank Milk

## Abstract

*Mycoplasma alkalescens* is an arginine-metabolizing mycoplasma, which has been found in association with mastitis and arthritis in cattle. Routine bacteriological examination of 17 bronchoalveolar lavage samples from calves with pneumonia in a single herd in Denmark, identified *M. alkalescens* in eight samples. The organism was found as a sole bacterilogical findings in five of the samples as well as in combination with *Mannheimia haemolytica*, *Haemophilus somni* and *Salmonella* Dublin. This is the first report of isolation of *M. alkalescens* in Denmark.

## Findings

*Mycoplasma alkalescens* is a bovine mycoplasma species, which was originally isolated from the nasal cavity of cattle in Australia [1]. Like many other mycoplasmas, *M. alkalescens* is a normal inhabitant of the upper respiratory tract, but it has also been associated with disease. *M. alkalescens* has mostly been implicated in mastitis in cattle. It has been isolated from bulk tank milk samples, as well as from outbreaks and sporadic cases of clinical mastitis [2–5]. Furthermore, *M. alkalescens* has been isolated from cases of severe arthritis, and its ability to induce joint lesions has been confirmed under experimental conditions [6, 7]. Rosenfeld & Hill [8] isolated *M. alkalescens* in pure culture from abomasum and lung of an aborted bovine foetus, while Lamm et al. [9] found *M. alkalescens* in association with otitis in calves. Finally, *M. alkalescens* has occasionally been found in association with disorders of the respiratory tract [10, 11].

Recently, 17 bronchoalveolar lavage samples from calves suffering from pneumonia in a single herd in Denmark were submitted, on two occasions, to the National Veterinary Institute for laboratory examinations. The samples were examined for the presence of bacterial pathogens, bovine respiratory mycoplasmas, as well as for the presence of bovine respiratory syncytial virus (BRSV), bovine coronavirus and parainfluenza-3 virus (PI-3). The samples were not examined for infectious bovine rhinotracheitis virus, as Denmark is considered free of this infection. Thereby, eight arginine-metabolizing mycoplasmas were isolated. In the present study the identification of the isolates as *M. alkalescens* is presented. This is the first report of isolation of this species in Denmark.

Bacteriological examination was performed according to standard laboratory procedures. Examination for BRSV, bovine coronavirus and PI-3 was performed using an indirect sandwich-ELISA assay [12]. Isolation of mycoplasmas was performed according to standard laboratory procedures using a Hayflick's type of medium enriched with arginine [13], with and without addition of 5% of rabbit hyperimmune antiserum against *Mycoplasma bovirhinis*. The isolates were filtered through 0.45 μm membrane filters (Millipore), cloned and submitted to serologic identification, which was performed by the disc growth inhibition test (DGI) with 6 mm filter paper discs prepared with rabbit hyperimmune antisera against *M. alkalescens* PG51^T^ (ATCC 29103; NCTC 10135), *Mycoplasma arginini* G230^T^ (ATCC 23838; NCTC 10129) and *Mycoplasma canadense* 275C^T^ (ATCC 29418; NCTC 10152), as well as by the indirect epi-immunofluorescence test (IF) on colonies on solid medium. Molecular identification of the isolates was performed by amplification and sequencing of their 16S rRNA genes by using universal primers [14].

Laboratory examinations revealed the presence of several bacterial pathogens in 15 of the 17 bronchoalveolar lavage samples, while no pathogenic bacteria were detected in the two remaining samples. *Pasteurella multocida*, *Mannheimia haemolytica*, *Histophilus somni* and *Salmonella* Dublin were found in three, three, one and one samples, respectively. *Mycoplasma bovirhinis* was found in five samples, while arginin-metabolizing mycoplasmas were found in eight samples. Two *P. multocida*, three *M. bovirhinis* and five arginin-metabolizing mycoplasma isolates were found as sole bacteriological findings in 10 respective samples. From five samples multiple bacterial species were isolated: *M. bovirhinis* was found once in combination with *P. multocida* and once in combination with *M. haemolytica*, while arginin-metabolizing mycoplasmas were found once in combination with *M. haemolytica*, once in combination with *S*. Dublin and once in combination with *M. haemolytica* and *H. somni*. No viral pathogens were detected in any of the analysed samples.

Further examinations on arginin-metabolizing mycoplasmas showed that all eight isolates could be serologically identified as *M. alkalescens* and that they were clearly different from the two other arginine-degrading species that are commonly found in cattle, *M. canadense* and *M. arginini* (Table 1). The reactions were clear-cut for the DGI tests showing approximately 3–5 mm broad zones of total or nearly total inhibition around the disc. In the IF test some minor cross reactions were noted when three of the isolates and the type strain of *M. alkalescens* PG51^T^ were tested with anti-*M. canadense* 275C^T^ hyperimmune serum. The 16S rDNA sequences of the analysed isolates were identical to each other and to the 16S rDNA sequence of the type strain *M. alkalescens* PG51^T^ (GenBank accession no. U44764; [15]), which corroborated the serological identification of the isolates as *M. alkalescens*.

**Table 1:** Serological identification of eight mycoplasma field isolates from cattle by DGI and IF test using type strain antisera against *Mycoplasma alkalescens*, *Mycoplasma arginini and Mycoplasma canadense*.

	Antiserum					
	*M. alkalescens* PG51^T^		*M. arginini* G230^T^		*M. canadense* 275C^T^	
Mycoplasma strains	DGI^a^	IF^b^	DGI^a^	IF^b^	DGI^a^	IF^b^
*M. alkalescens* PG51^T^	5	+	0	-	0	?
Field strain MK19/02	≥ 3	+	0	-	0	-
Field strain MK20/02	≥ 3	+	0	-	0	?
Field strain MK21/02	≥ 3	+	0	-	0	-
Field strain MK49/02*	≥ 3	+	0	-	0	?
Field strain MK51/02	≥ 3	+	0	-	0	-
Field strain MK52/02*	≥ 3	+	0	-	0	-
Field strain MK53/02	≥ 3	+	0	-	0	-
Field strain MK56/02*	≥ 3	+	0	-	0	?
*M. arginini* G230^T^	0	-	5	+	0	-
*M. canadense* 275C^T^	0	-	0	-	5	+

^a^ zone of inhibition in mm; ^b^ distinct FITC colour of stained colonies; ? inconclusive results; * examined by 16S rDNA sequencing.

Previous studies have shown that *H. somni*, *M. haemolytica* and *P. multocida* are the bacteria that are most commonly associated with bronchopneumonia in calves in Denmark, although they are also part of the normal bacterial flora of the respiratory tract [16, 17]. Also, *S*. Dublin, which is predominantly found in cattle, is capable of causing pneumonia in calves. Isolation of these bacterial species in the submitted samples most likely indicate their role as the primary cause of respiratory disease in the herd, probably in combination with unidentified environmental factors. In addition to the bacteria, *M. bovirhinis* and *M. alkalescens* were also isolated. *M. bovirhinis* is commonly found as a part of the normal respiratory tract microflora of cattle and is not considered to be pathogenic. *M. alkalescens*, however, has been found in association with disorders of the respiratory tract but its role as a respiratory pathogen remains equivocal. Experimental infections have demonstrated that *M. alkalescens* has the ability to colonize lung tissue [18], but the two strains used in the study apparently failed to produce pneumonia [18]. In the present study, we found *M. alkalescens* in pure culture in five of the analysed bronchoalveolar lavage samples. This finding is, however, not sufficient to warrant a role of *M. alkalescens* as a cause of bronchopneumonia, since we also found two samples containing only a non-pathogenic *M. bovirhinis*, and two samples without any bacterial or viral pathogens, despite the fact that they derived from calves with clinical signs of a respiratory disease. The failure to detect respiratory tract pathogens in these samples may be due to i.e., their absence in a particular disease stadium when sampling took a place or due to antibiotic treatment. Taking into the consideration the overall bacteriological findings of this study, it seems likely that *M. alkalescens* may have had a role either as a secondary invader or as an opportunistic pathogen rather than suggesting a causal role of the organism in the pneumonia complex.

*M. alkalescens* is regarded as one of the most common causative agents of mastitis [19]. So, with demonstration of the organism in a Danish cattle herd, a further member of *Mycoplasma* is added to the group of bovine mastitis-inducing microorganisms in Denmark. The only mycoplasma species isolated from clinical outbreaks of mastitis in Denmark so far has been *Mycoplasma bovis* [20], while other mastitis-inducing species, *Mycoplasma bovigenitalium* and *M. canadense*, have been isolated only from the respiratory and the genital tract and semen samples [21]. Further investigations are needed in order to determine the prevalence of *M. alkalescens* in the Danish cattle population and, indeed, to draw a firm conclusions on its importance in disease conditions other than mastitis.
